# Impact of chosen cutoff on response rate differences between selective serotonin reuptake inhibitors and placebo

**DOI:** 10.1038/s41398-022-01882-5

**Published:** 2022-04-14

**Authors:** Alexander Lisinski, Fredrik Hieronymus, Staffan Nilsson, Elias Eriksson

**Affiliations:** 1 Department of Pharmacology, Institute of Neuroscience and Physiology, Sahlgrenska Academy, University of Gothenburg, Gothenburg, Sweden; 2 Department of Clinical Medicine, Aarhus University, Aarhus, Denmark; 3 Department of Pathology and Genetics, Institute of Biomedicine, Sahlgrenska Academy, University of Gothenburg, Gothenburg, Sweden; 4 Institute of Mathematical Sciences, Chalmers University of Technology, Gothenburg, Sweden

**Keywords:** Depression, Clinical pharmacology

## Abstract

Response defined as a 50% reduction in the sum score of the Hamilton Depression Rating Scale (HDRS-17-sum) is often used to assess the efficacy of antidepressants. Critics have, however, argued that dichotomising ratings with a cutoff close to the median may lead to scores clustering on either side, the result being inflation of miniscule drug-placebo differences. Using pooled patient-level data sets from trials of three selective serotonin reuptake inhibitors (SSRIs) (citalopram, paroxetine and sertraline) (*n* = 7909), and from similar trials of duloxetine (*n* = 3478), we thus assessed the impact of different cutoffs on response rates. Response criteria were based on (i) HDRS-17-sum, (ii) the sum score of the HDRS-6 subscale (HDRS-6-sum) and (iii) the depressed mood item. The separation between SSRI and placebo with respect to response rates increased when HDRS-17-sum was replaced by HDRS-6-sum or depressed mood as effect parameter and was markedly dependent on SSRI dose. With the exception of extreme cutoff values, differences in response rates were largely similar regardless of where the cutoff was placed, and also not markedly changed by the exclusion of subjects close to the selected cutoff (e.g., ±10%). The observation of similar response rate differences between active drugs and placebo for different cutoffs was corroborated by the analysis of duloxetine data. In conclusion, the suggestion that using a cutoff close to the median when defining response has markedly overestimated the separation between antidepressants and placebo may be discarded.

## Introduction

A 50% reduction in the total sum of the 17 items comprising the Hamilton Depression Rating Scale (HDRS-17-sum) has been a common definition of response in trials assessing the efficacy of selective serotonin reuptake inhibitors (SSRIs) and other antidepressants. Critics have, however, argued that selecting a cutoff close to the median of the endpoint score distribution curve may result in significant differences in response rates between groups also when the actual differences in mean rating are miniscule and clinically irrelevant [1]. Indeed, if, for example, all antidepressant-treated patients improve by 50% and all those treated with placebo by 49%, the resultant 100% difference in response rates would be statistically highly significant but clinically unimportant. Conversely, it has been argued that using HDRS-17-sum as an effect parameter may underestimate the actual antidepressant effects of SSRIs, one reason being that several items included in this scale may capture common side effects of these drugs [2–4].

Using pooled patient-level data from 28 trials comparing an SSRI with placebo, we explored the impact of the placement of dichotomised cutoffs on the SSRI versus placebo separation. To this end, three different outcome measures were used: HDRS-17-sum, the sum score of the unidimensional HDRS-6 subscale including six core symptoms of depression from the HDRS (HDRS-6-sum) [5], and the depressed mood item [2]. To corroborate the results obtained using the SSRI data, corresponding analyses were performed using patient-level data from 13 trials comparing serotonin- and noradrenaline reuptake inhibitor (SNRI), duloxetine, to placebo. To further address concerns that subjects clustering close to but on either side of the cutoff might inflate negligible mean drug-placebo differences [1], we also used the data from the SSRI trials to assess the effect of excluding subjects closest to the cutoffs (e.g., ±10%) from the analyses. Finally, we assessed response and remission rates for what we have previously [6] suggested to be optimal and suboptimal SSRI doses, respectively.

## Materials and methods

### Data acquisition

Patient-level data from 28 industry-sponsored, placebo-controlled, acute phase trials for adults with major depression using the HDRS-17 as symptom inventory were obtained for citalopram (Lundbeck, Valby, Denmark), paroxetine (GlaxoSmithKline, Brentford, UK) and sertraline (Pfizer, New York, NY, USA). In two of the paroxetine studies and one sertraline study, fluoxetine was used as active control; while these patients were also included, those treated with non-SSRI comparators were not. To corroborate results obtained using data from the SSRI trials, we also analysed patient-level data from 13 trials comparing duloxetine to placebo (Lilly, Indianapolis, IN, USA). SSRI comparators (escitalopram, fluoxetine and paroxetine) were excluded. Both these data sets have been previously described in greater detail [7, 8].

### Statistical analyses

To visualise the separation of SSRIs and placebo at different cutoffs for percentage reduction from baseline, we plotted the cumulative proportions of SSRI- and placebo-treated patients whose remaining symptoms at endpoint corresponded to every 5% fraction of their baseline score for HDRS-17-sum and HDRS-6-sum, respectively. For depressed mood, comprising only four possible levels, change scores rather than % reduction were plotted. For simplified visualisation, patients who were unchanged or had deteriorated during treatment were all included in one data point as if they had displayed 100% of baseline scores at endpoint for HDRS-17-sum and HDRS-6-sum or had displayed no change for depressed mood.

Corresponding visualisations were produced for endpoint scores with respect to the HDRS-17-sum, HDRS-6-sum and the depressed mood item; for visualisation purposes, maximum scores were capped at 40 points for HDRS-17-sum (range 0–52) and at 20 points for HDRS-6-sum (range 0–22); subjects displaying higher scores being included in the highest visualised score.

All visualisations were performed on patients with at least one pre- and post-baseline HDRS-17 measure in both the intention-to-treat (ITT) last observation carried forward (LOCF) population and in the observed cases (OC) population. Week 6 was used as the primary endpoint since most studies had an evaluation at that time. For studies with no week 6 observation, the closest observation was used (week 4 for five studies and week 8 for one study; see Supplementary Table 1).

Similar visualisations were undertaken also with respect to the duloxetine versus placebo trials. For these data, week 8 was used as endpoint observation; if week 8 data were missing, data from the closest observation was used (week 7 for one study and week 9 for two studies; Supplementary Table 2).

We also used data from the SSRI trials to model odds ratios (ORs) for response (SSRI versus placebo) for all possible 10% intervals of HDRS-17-sum and HDRS-6-sum reductions from baseline, and also for all possible change scores for depressed mood. Similar analyses were undertaken with respect to endpoint scores in the range of 0–10 points for HDRS-17-sum and HDRS-6-sum and 0–4 points for depressed mood, hence including cutoffs commonly used to define remission [9–15]. This was done using a generalised mixed model which included treatment, time (week) and trial as fixed factors as well as the interaction between treatment and time. The baseline rating on the outcome measure in question (HDRS-17-sum, HDRS-6-sum, or depressed mood) was included as a covariate. The model utilised a binary distribution with a logit link, the Kenward–Roger method was used to estimate denominator degrees of freedom and an unstructured covariance matrix was used to model within-patient errors. All time points between week 1 and endpoint were included in the model but only results at week 6 are reported. If the models did not converge, we first excluded the observation at week 5 (which was available only for a minority of studies) and then, if needed, the trial fixed factor. If convergence was still not attained, unmodelled LOCF numbers were used with statistical significance analysed using the chi-square test.

To explore whether differences between-treatment groups with respect to response rates might be explained by patients clustering close to, but on either side of, any particular cutoff, we also used the SSRI data to model ORs for established response or remission criteria [9, 10] after removing patients with endpoint scores just above and/or below the cutoff in question, i.e., ±5% and ±10% for percentage reduction-based outcomes and ±1 and ±2 points for endpoint score cutoffs.

Finally, to assess the impact of SSRI dose on dichotomous definitions of response and remission, we pooled data from those trials that had assessed fixed SSRI doses, hence excluding flexible-dose trials (Supplementary Table 1). Based on a previous report based on the same data set [6], suboptimal doses were defined as citalopram 10–20 mg, sertraline 50 mg, paroxetine 10 mg and optimal doses as citalopram 40–60 mg, sertraline 100–400 mg and paroxetine 20–40 mg. Paroxetine controlled-release doses of 12.5 mg and 25 mg were assumed to correspond to 10 mg and 20 mg of paroxetine immediate-release, respectively. A three-level variable coding for placebo, suboptimal SSRI dose and optimal SSRI dose, respectively, replaced the treatment variable in a generalised linear mixed model otherwise identical to the initial model used to obtain OSs for all possible 10% intervals.

All analyses were performed using SAS version 9.4 (SAS Institute, Cary, NC, USA). For the duloxetine data, remote desktop access to the Clinical Trial Data Transparency environment was provided through SAS Solutions OnDemand by the Clinical Study Data Request website. All *p* values were two-tailed and the significance level was set at *α* = 0.05.

### Ethics

The Regional Ethics Review Board of Gothenburg, Sweden, issued an advisory opinion stating no objection to the conduct of post hoc analyses of clinical trial data. As data were anonymised, informed patient consent was waived.

## Results

**Fig. 1:**
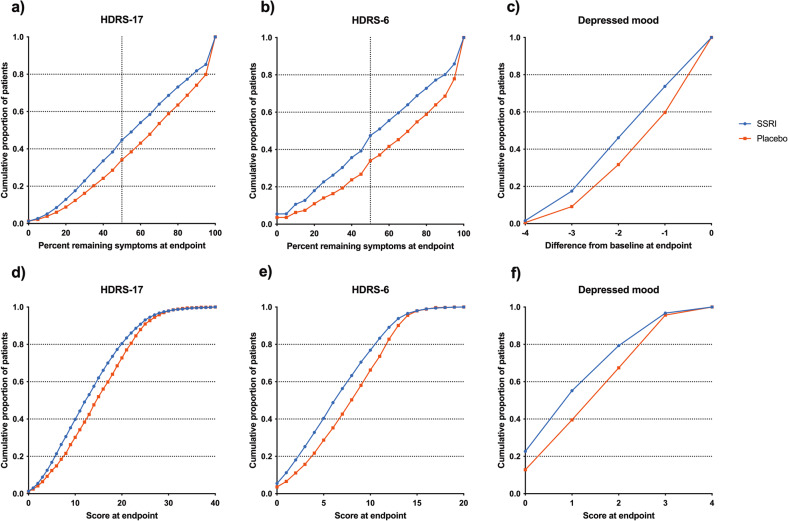
SSRI and placebo response rates. Cumulative proportions of SSRI- and placebo-treated patients scoring on or below each 5% fraction of baseline scores at endpoint are shown for HDRS-17-sum in **a** and for HDRS-6-sum in **b**. Cumulative proportions of SSRI- and placebo-treated patients reporting different score reductions with respect to depressed mood are shown in **c**. Corresponding data but for each endpoint score are displayed in **d** (HDRS-17-sum), **e** (HDRS-6) and **f** depressed mood. Shown is the ITT-LOCF population. *n* = 5424 (SSRI) and 2485 (placebo). Patients deteriorating during treatment were coded as having an endpoint fraction of 100% **a**–**b** or a change score of zero **c**. The share of patients deteriorating was **a** SSRIs 8.8%; placebo 12.4%, **b** SSRIs 7.4%; placebo 11.9%, **c** SSRIs 3.7%; placebo 7.0%. Endpoint scores were capped at 40 points (**d**) and 20 points (**e**), respectively. The share of patients scoring above these thresholds was **d** SSRIs 0.13%, placebo 0.04%, **e** SSRIs 0.02%, placebo 0.00%.

In total, 7909 patients from SSRI versus placebo trials with a post-baseline HDRS-17 observation were included in the analyses. Inspection of the cumulative plots for relative score reductions in SSRI- and placebo-treated patients, respectively, revealed largely parallel lines across a wide range of possible outcomes (Fig. 1)‚ with the exception of very low (≤20%) and very high (≤90%) cutoffs, SSRIs thus separated from placebo with roughly the same absolute magnitude regardless of cutoff. The SSRI versus placebo separation was numerically greater for HDRS-6-sum and depressed mood than for HDRS-17-sum.

Cumulative plots for endpoint scores were similar to those for percentage reduction and change scores. Drug versus placebo differences was again most prominent when assessed using HDRS-6-sum or the depressed mood item as effect parameter, but was largely independent of the chosen cutoff, except, with respect to HDRS-17-sum and HDRS-6-sum, for values near the bottom or the top of the range. For depressed mood, only values near the top of the range showed less SSRI versus placebo separation. The plots for the observed cases population displayed a similar pattern (Supplementary fig. 1).

Similar visualisations as those produced for the SSRI trials were created also for the duloxetine trials (number of subjects: 3478) and are displayed in Supplementary figs. 2 and 3. Again the placement of the cutoff for dichotomisation was found to exert no major impact on the separation of active drug versus placebo.

The modelled analyses confirmed that the separation between SSRIs and placebo on relative measures of improvement was largely independent of the cutoff used except for at very high and very low values (Table 1). ORs were generally higher for HDRS-6-sum and depressed mood than for HDRS-17-sum. Removing patients close to the commonly used cutoffs (±5 or ±10%) did not markedly impact ORs or absolute differences. Similar patterns were observed for endpoint score-based cutoffs (Table 2).

**Table 1:** Modelled proportions of responders defined using different cutoffs in SSRI- and placebo-treated patients; *n* = 5424 (SSRI) and 2485 (placebo).

	Modelled proportions of responders				
HDRS-17-sum: remaining proportion of symptoms	SSRI	Placebo	Difference	*p*	Odds ratio (95% Cl)
0%	1.6%	1.6%	0.0%	0.99	1.00 (0.62–1.61)
≤10%	6.6%	5.0%	1.6%	0.03	1.34 (1.03–1.74)
≤20%	15.5%	10.0%	5.5%	<0.0001	1.65 (1.37–1.99)
≤20% (excluding 15–25%)	11.7%	8.2%	3.5%	0.0002	1.48 (1.20–1.82)
≤20% (excluding 10–30%)	9.2%	6.3%	2.8%	0.0006	1.49 (1.19–1.87)
≤30%	28.7%	20.0%	8.7%	<0.0001	1.61 (1.39–1.86)
≤40%	42.3%	29.7%	12.6%	<0.0001	1.73 (1.52–1.97)
≤50%	54.6%	42.1%	12.5%	<0.0001	1.65 (1.47–1.87)
≤50% (excluding 45–55%)	52.5%	39.1%	13.4%	<0.0001	1.72 (1.52–1.95)
≤50% (excluding 40–60%)	51.9%	37.9%	14.0%	<0.0001	1.77 (1.55–2.01)
≤60%	65.7%	52.4%	13.3%	<0.0001	1.74 (1.55–1.96)
≤70%	74.8%	63.4%	11.5%	<0.0001	1.72 (1.52–1.94)
≤80%	82.7%	72.2%	10.5%	<0.0001	1.84 (1.61–2.11)
≤90%	89.3%	81.2%	8.1%	<0.0001	1.93 (1.65–2.26)
≤100%	95.1%	91.1%	4.0%	<0.0001	1.91 (1.54–2.38)
**HDRS-6-sum: remaining proportion of symptoms**	**SSRI**	**Placebo**	**Difference**	***p***	**Odds ratio (95% Cl)**
0%	6.7%	4.6%	2.2%	0.003	1.50 (1.15–1.97)
≤10%	13.5%	7.9%	5.6%	<0.0001	1.82 (1.48–2.23)
≤20%	19.1%	11.5%	7.6%	<0.0001	1.82 (1.54–2.16)
≤20% (excluding 15–25%)	15.8%	9.3%	6.5%	<0.0001	1.84 (1.52–2.22)
≤20% (excluding 10–30%)	14.0%	8.3%	5.6%	<0.0001	1.78 (1.46–2.17)
≤30%	30.7%	19.2%	11.5%	<0.0001	1.86 (1.61–2.15)
≤40%	43.2%	28.9%	14.3%	<0.0001	1.87 (1.65–2.13)
≤50%	58.3%	42.1%	16.3%	<0.0001	1.93 (1.71–2.18)
≤50% (excluding 45–55%)	54.5%	38.2%	16.3%	<0.0001	1.94 (1.71–2.20)
≤50% (excluding 40–60%)	54.2%	37.3%	16.9%	<0.0001	1.99 (1.75–2.26)
≤60%	66.7%	50.4%	16.3%	<0.0001	1.97 (1.75–2.22)
≤70%	74.9%	59.0%	15.9%	<0.0001	2.07 (1.83–2.34)
≤80%	82.1%	67.4%	14.8%	<0.0001	2.22 (1.95–2.54)
≤90%	88.0%	76.5%	11.5%	<0.0001	2.26 (1.95–2.61)
≤100%	96.1%	92.5%	3.5%	<0.0001	1.97 (1.56–2.49)
**Depressed mood: change from baseline**	**SSRI**	**Placebo**	**Difference**	***p***	**Odds ratio (95% Cl)**
−4	1.6%^*^	0.6%^*^	1.0%^*^	0.0002^*^	2.72 (1.57–4.70)^*^
≤−3	14.9%	7.8%	7.2%	<0.0001	2.08 (1.75–2.47)
≤−2	55.8%	39.1%	16.6%	<0.0001	1.96 (1.73–2.22)
≤−1	82.7%	69.1%	13.6%	<0.001	2.13 (1.87–2.44)
≤0	98.7%	96.7%	2.1%	<0.0001	2.73 (2.00–3.73)

^*^Unmodelled LOCF population.

**Table 2:** Modelled proportions of responders defined using different cutoffs with respect to endpoint scores in SSRI- and placebo-treated patients; *n* = 5424 (SSRI) and 2485 (placebo).

	Modelled proportions of responders				
HDRS-17 endpoint score	SSRI	Placebo	Difference	*p*	Odds ratio (95% Cl)
0 p	1.3%^*^	1.3%^*^	0.0%^*^	0.87^*^	1.04 (0.68–1.58)^*^
≤1 p	3.8%	3.2%	0.7%	0.25	1.22 (0.87–1.71)
≤2 p	6.9%	5.0%	1.9%	0.01	1.40 (1.07–1.82)
≤3 p	10.9%	7.4%	3.5%	0.0002	1.53 (1.26–1.90)
≤4 p	14.6%	10.4%	4.2%	<0.0001	1.47 (1.22–1.78)
≤5 p	20.5%	14.6%	5.9%	<0.0001	1.51 (1.28–1.78)
≤6 p	26.3%	17.6%	8.6%	<0.0001	1.66 (1.43–1.94)
≤7 p	33.1%	22.3%	10.8%	<0.0001	1.72 (1.50–1.99)
≤7 p (excluding 6–8 p)	24.9%	17.1%	7.9%	<0.0001	1.61 (1.38–1.89)
≤7 p (excluding 5–9 p)	22.4%	15.3%	7.0%	<0.0001	1.59 (1.35–1.88)
≤8 p	37.8%	25.5%	12.2%	<0.0001	1.77 (1.55–2.03)
≤9 p	43.2%	31.6%	11.6%	<0.0001	1.65 (1.45–1.87)
≤10 p	48.8%	36.6%	12.2%	<0.0001	1.65 (1.46–1.87)
**HDRS-6 endpoint score**	**SSRI**	**Placebo**	**Difference**	***p***	**Odds ratio (95% Cl)**
0 p	6.7%	4.6%	2.2%	0.003	1.51 (1.15–1.98)
≤1 p	14.0%	7.9%	6.1%	<0.0001	1.90 (1.55–2.34)
≤2 p	19.8%	11.8%	8.0%	<0.0001	1.85 (1.56–2.19)
≤3 p	29.6%	18.5%	11.2%	<0.0001	1.86 (1.60–2.16)
≤4 p	40.0%	26.3%	13.8%	<0.0001	1.88 (1.64–2.15)
≤4 p (excluding 3–5 p)	30.8%	18.5%	12.3%	<0.0001	1.96 (1.68–2.29)
≤4 p (excluding 2–6 p)	27.4%	16.2%	11.3%	<0.0001	1.96 (1.66–2.31)
≤5 p	49.7%	35.2%	14.5%	<0.0001	1.82 (1.60–2.06)
≤6 p	59.2%	42.4%	16.8%	<0.0001	1.97 (1.74–2.23)
≤7 p	67.4%	51.1%	16.3%	<0.0001	1.98 (1.74–2.24)
≤8 p	75.3%	60.2%	15.0%	<0.0001	2.01 (1.76–2.29)
≤9 p	82.5%	68.0%	14.5%	<0.0001	2.21 (1.92–2.55)
≤10 p	87.8%	76.2%	11.6%	<0.0001	2.25 (1.92–2.63)
**Depressed mood endpoint score**	**SSRI**	**Placebo**	**Difference**	***p***	**Odds ratio (95% Cl)**
0 p	27.1%	15.7%	11.4%	<0.0001	2.00 (1.70–2.34)
≤1 p	66.4%	48.4%	18.0%	<0.0001	2.10 (1.86–2.39)
≤2 p	89.3%	77.6%	11.7%	<0.0001	2.41 (2.05–2.82)
≤3 p	99.6%	99.0%	0.6%	0.04	2.27 (1.05–4.92)

^*^Unmodelled LOCF population.

For all tested cutoffs, drug versus placebo differences were larger for optimal SSRI doses than for suboptimal ones (Table 3). Differences between doses were also larger for most comparisons when using outcome measures based on HDRS-6-sum or depressed mood rather than on HDRS-17-sum.

**Table 3:** Effect of SSRI dose on modelled proportions of responders and remitters defined using commonly proposed cutoffs based on remaining symptoms in SSRI- and placebo-treated patients; *n* = 876 (suboptimal doses); *n* = 1299 (optimal doses); and *n* = 753 (placebo).

		Suboptimal doses				Optimal doses			
Criterion	Placebo	SSRI	Difference	*p*	Odds ratio (95% Cl)	SSRI	Difference	*p*	Odds ratio (95% Cl)
≤50% HDRS-17	43.1%	51.0%	7.9%	0.02	1.38 (1.06–1.78)	61.2%	18.1%	<0.0001	2.08 (1.62–2.67)
≤50% HDRS-6	43.7%	54.2%	10.5%	0.001	1.53 (1.18–1.97)	66.0%	22.3%	<0.0001	2.50 (1.95–3.21)
≤80% HDRS-17	11.0%	17.0%	6.0%	0.007	1.65 (1.15–2.39)	21.2%	10.1%	<0.0001	2.17 (1.53–3.07)
≤80% HDRS-6	11.4%	17.1%	5.6%	0.006	1.60 (1.14–2.22)	20.6%	9.2%	<0.0001	2.01 (1.46–2.77)
≤7 HDRS-17	24.6%	33.1%	8.5%	0.005	1.52 (1.14–2.03)	39.2%	14.7%	<0.0001	1.98 (1.51–2.62)
≤4 HDRS-6	27.4%	38.5%	11.2%	0.0003	1.66 (1.26–2.19)	45.1%	17.8%	<0.0001	2.18 (1.67–2.85)
0 Depressed mood	15.4%	26.4%	11.0%	<0.0001	1.97 (1.41–2.76)	34.3%	18.8%	<0.0001	2.86 (2.07–3.94)

Suboptimal doses defined as citalopram 10–20 mg, sertraline 50 mg, paroxetine 10 mg; optimal doses as citalopram 30–40 mg, sertraline 100–400 mg, paroxetine 20–40 mg. These data have been partly reported in a previous publication [6].

## Discussion

In this report, we rebut the claim [1] that differences between SSRIs and placebo with respect to response rates be inflated due to the choice of a cutoff for defining response near the median; the discrimination of treatments in this regard was hence largely independent of the placement of the cutoff. Moreover, in line with previous studies based on mean ratings, we show the separation between active treatment and placebo also for dichotomous responses to be larger when using HDRS-6-sum or the individual item depressed mood rather than HDRS-17-sum as effect parameter. Finally, between-treatment differences were larger when including optimal SSRI doses only. We hence conclude that differences between SSRIs and placebo with respect to their ability to induce response or remission in previous meta-analyses [16] may have been underrated by the use of an insensitive measure, i.e., HDRS-17-sum, and by the inclusion of suboptimal doses, but that the definition of response or remission is less consequential for the outcome. The choice of response-defining cutoff not exerting a major impact on the separation between active treatment and placebo was confirmed in an independent sample of trials comparing duloxetine and placebo.

The lack of impact of the placement of the cutoff is visually illustrated by the largely parallel lines over most of the range with respect to the cumulative distribution of symptom reduction scores (Fig. 1, Supplementary Figs. 1–3). In line with this, and further demonstrating that differences in response rates are not caused by subjects in the respective treatment groups clustering near but on either side of a particular cutoff, exclusion of subjects close to common cutoffs did not markedly impact response rates (Tables 1 and 2). Instead, differences between groups with respect to common definitions of response and remission primarily reflect differences in distribution with respect to low remaining symptom scores, where there is a predominance of subjects in the active treatment group and with respect to high remaining symptom scores, where there is a corresponding clustering of placebo-treated subjects [17]. The placement of the cutoff is hence largely irrelevant as long as it is not placed near the extreme values (i.e., where the lines in the figures are not parallel).

We [2, 8] and others [18, 19] have previously reported that using HDRS-17-sum as an effect parameter may make SSRIs and SNRIs appear less effective than they actually are in reducing core symptoms of depression such as depressed mood. Although the separation of active drug from placebo with respect to mean symptom rating has thus been shown more robust when using HDRS-6-sum instead of HDRS-17-sum as a measure, we now report that this difference, not unexpectedly, also translates into corresponding differences with respect to ORs for dichotomous criteria of response and remission. A similar observation (based on the same SSRI data set) was previously reported for the depressed mood item [6] and was here extended to include additional definitions of response or remission.

Of note is that the separation between active drug and placebo with respect to the proportion of subjects displaying very low remaining endpoint scores as well as proportions of baseline scores was substantial when assessed using the shorter and unidimensional HDRS-6 subscale but less so when using HDRS-17-sum. Since healthy volunteers on average score about three points on the HDRS [20], since particularly some of the items not included in the HDRS-6 subscale may capture side effects of active treatment [4, 19], and since residual symptoms of depression usually remain after only 6 weeks of treatment also in responders [21, 22], it is not surprising that only a few subjects displayed very low HDRS-17-sum scores also in the actively treated group.

A factor possibly impacting efficacy is the dose of active treatment. While the dose–response curve for the SSRIs have often been described as flat [23, 24], and while most trial-based meta-analyses have included all SSRI doses as if they be equally effective [16, 25], using the same data set as in the present study, we have previously reported [6] 50 mg of sertraline, 20 mg of citalopram and 10 mg of paroxetine to demonstrate lower efficacy than higher doses of the same compounds both in terms of mean symptom rating and dichotomous assessment of response and remission; similar conclusions have subsequently been advocated also by others [26]. We have now expanded these analyses to comprise additional dichotomous outcome measures, again yielding consistently higher response and remission rates for optimal doses as compared to what is obtained with doses at the lower end of the dosing interval. Of note is the impressive difference between optimal doses of SSRIs versus placebo, e.g., with respect to obtaining a 50% reduction on HDRS-17-sum or HDRS-6-sum (~60% versus 40%).

This study has some limitations. First, HDRS scores might be inflated at baseline [27], which may result in an artificial symptom reduction, regardless of treatment, when the same instrument is used for inclusion and evaluation of response. Second, methodological problems related, e.g., to poor compliance [28, 29] or to overly liberal recruitment of participants [30], which may also reduce the apparent difference between active drug and placebo, are bound to impact not only the outcome of the individual trials but also that of post hoc analyses. Third, the results presented may not necessarily translate to antidepressants with other mechanisms of action or to subjects below the age of eighteen.

In conclusion, this report rebuts the previous claim that differences between antidepressants and placebo with respect to response rates be inflated by the common use of a cutoff (50%) close to the median with subjects clustering on either side. Instead, we report response rate differences consistently larger for higher SSRI doses and for outcomes based on the unidimensional HDRS-6 subscale or the single item depressed mood, indicating that previous meta-analyses in this field may have, on the contrary, deflated the differences between SSRIs and placebo with respect to response rates by including suboptimal doses and applying an insensitive outcome measure.

## Supplementary information

Supplementary information

## Data Availability

Qualified researchers can apply to obtain the data underlying this publication through the respective pharmaceutical companies. Supplementary information is available on MP’s website.

## Code availability

The computer code used to generate the results published here can be obtained by contacting the authors.
