# Supplementary material for: Impact of chosen cutoff on response rate differences between selective serotonin reuptake inhibitors and placebo
Source: Transl Psychiatry. 2022 Apr 14;12:160. doi: 10.1038/s41398-022-01882-5 (PMC9010419; doi:10.1038/s41398-022-01882-5)
Supplement: Supplementary file 1 — Supplementary information [file 41398_2022_1882_MOESM1_ESM.docx]

**Lisinski et al: Impact of chosen cut-off on response rate differences between selective serotonin reuptake inhibitors and placebo: Supplementary information**

*Supplementary Results Tables and Figures*

**Supplementary table 1:** Included SSRI trials.

**Supplementary table 2:** Included duloxetine trials.

**Supplementary figure 1:** Cumulative proportions of SSRI- and placebo-treated patients scoring on or below each 5% fraction of baseline scores, or on or below each possible endpoint score: observed cases population.

**Supplementary figure 2:** Cumulative proportions of duloxetine- and placebo-treated patients scoring on or below each 5% fraction of baseline scores, or on or below each possible endpoint score: intention-to-treat last observation carried forward population.

**Supplementary figure 3:** Cumulative proportions of duloxetine- and placebo-treated patients scoring on or below each 5% fraction of baseline scores, or on or below each possible endpoint score: observed cases population.

**Supplementary table 1** Included SSRI trials.

| **Protocol** | **Sponsor** | **Groups** | **N (ITT)** | **N (week 6)** | **Length (weeks)** | **Fixed dose** |
| --- | --- | --- | --- | --- | --- | --- |
| 1 | GSK | PRX IR, PLA | 48 | 27^*^ | 4 | - |
| 2 | GSK | PRX IR, PLA | 324 | 191 | 6 | - |
| 3 | GSK | PRX IR, PLA | 468 | 244 | 6 | - |
| 9 | GSK | PRX IR, PLA | 450 | 292 | 12 | 10, 20, 30, 40 mg |
| 115 | GSK | FLX, PRX IR, PLA | 657 | 487 | 12 | - |
| 128 | GSK | FLX, PRX IR, PLA | 825 | 612 | 12 | - |
| 251 | GSK | PRX IR, PLA | 243 | 181 | 8 | - |
| 274 | GSK | PRX IR, PLA | 41 | 30^*^ | 4 | 30 mg |
| 276 | GSK | PRX IR, PLA | 41 | 17 | 6 | 30 mg |
| 279 | GSK | PRX IR, PLA | 29 | 15 | 6 | 30 mg |
| 448 | GSK | PRX CR, PRX IR, PLA | 307 | 244 | 12 | - |
| 449 | GSK | PRX CR, PRX IR, PLA | 328 | 270 | 12 | - |
| 487 | GSK | PRX CR, PRX IR, PLA | 314 | 269 | 12 | - |
| 810 | GSK | PRX CR, PLA | 436 | 371 | 8 | 12.5, 25 mg |
| 874 | GSK | PRX CR, PLA | 512 | 392^†^ | 10 | 12.5, 25 mg |
| 101 | Pfizer | SER, PLA | 120 | 66^*^ | 4 | 50, 100, 200, 400 mg |
| 103 | Pfizer | SER, PLA | 347 | 199 | 6 | 50, 100, 200 mg |
| 104 | Pfizer | SER, PLA | 283 | 202 | 8 | - |
| 109 | Pfizer | SER, PLA | 207 | 141 | 8 | - |
| 111 | Pfizer | FLX, SER, PLA | 319 | 226 | 8 | - |
| 238 | Pfizer | SER, PLA | 190 | 131 | 10 | - |
| 240 | Pfizer | SER, PLA | 69 | 39 | 10 | - |
| 247 | Pfizer | SER, PLA | 81 | 44 | 10 | - |
| 310 | Pfizer | SER, PLA | 168 | 128^*^ | 4 | 50, 100, 200, 400 mg |
| 315 | Pfizer | SER, PLA | 149 | 102 | 8 | - |
| 85 | Lundbeck | CIT, PLA | 169 | 99^*^ | 4 | - |
| 89303 | Lundbeck | CIT, PLA | 193 | 146 | 6 | 20, 40 mg |
| 91206 | Lundbeck | CIT, PLA | 591 | 439 | 6 | 10, 20, 40, 60 mg |

^*^Observations at week 4 are used. ^†^Observations at week 8 are used. CIT = citalopram; FLX = fluoxetine; ITT = intention to treat; PLA = placebo; PRX CR = paroxetine controlled release; PRX IR = paroxetine immediate release; SER = sertraline.

**Supplementary table 2** Included duloxetine trials.

| **Study** | **Sponsor** | **Groups^*^** | **N (ITT)** | **N (week 8)** | **Length (weeks)** |
| --- | --- | --- | --- | --- | --- |
| *HMAQa* | Lilly | DLX, PLA | 109 | 71 | 8 |
| *HMAQb* | Lilly | DLX, PLA | 121 | 81 | 8 |
| *HMATa* | Lilly | DLX, PLA | 195 | 138 | 8 |
| *HMATb* | Lilly | DLX, PLA | 192 | 124 | 8 |
| *HMAYa* | Lilly | DLX, PLA | 264 | 231 | 8 |
| *HMAYb* | Lilly | DLX, PLA | 283 | 259 | 8 |
| *HMBHa* | Lilly | DLX, PLA | 236 | 173^†^ | 9 |
| *HMBHb* | Lilly | DLX, PLA | 259 | 171^†^ | 9 |
| *HMBV* | Lilly | DLX, PLA | 249 | 203 | 8 |
| *HMCB* | Lilly | DLX, PLA | 268 | 192^‡^ | 7 |
| *HMCR* | Lilly | DLX, PLA | 291 | 214 | 8 |
| *HMFA* | Lilly | DLX, PLA | 278 | 218 | 12 |
| *HMFS* | Lilly | DLX, PLA | 733 | 632 | 8 |

^*^SSRI comparators are excluded. ^†^Observations at week 9 are used. ^‡^Observations at week 7 are used. DLX = duloxetine; ESC = escitalopram; FLX = fluoxetine; ITT = intention to treat; PLA = placebo; PRX = paroxetine.

**Supplementary figure 1** Cumulative proportions of SSRI- and placebo-treated patients scoring on or below each 5% fraction of baseline scores at endpoint are shown for HDRS-17-sum in (a) and for HDRS-6-sum in (b). Cumulative proportions of SSRI- and placebo-treated patients reporting different score reductions with respect to depressed mood are shown in (c). Corresponding data but for each endpoint score are displayed in (d) (HDRS-17-sum), (e) (HDRS-6) and (f) depressed mood (f). Patients deteriorating during treatment were coded as having an endpoint fraction of 100% (a-b) or a change score of zero (c). The share of patients deteriorating was (a) SSRIs 4.0%; placebo 7.3%, (b) SSRIs 4.0%; placebo 7.1%, (c) SSRIs 2.0%; placebo 5.2%. Endpoint scores were capped at 40 points (d) and 20 points (e), respectively. The share of patients scoring above these thresholds was (d) SSRIs 0.00%, placebo 0.06%, (e) SSRIs 0.00%, placebo 0.00%. Shown is the observed cases population; n=3 871 (SSRI) and n=1 733 (placebo).

**Supplementary figure 2** Cumulative proportions of duloxetine- and placebo-treated patients scoring on or below each 5% fraction of baseline scores at endpoint are shown for HDRS-17-sum in (a) and for HDRS-6-sum in (b). Cumulative proportions of duloxetine- and placebo-treated patients reporting different score reductions with respect to depressed mood are shown in (c). Corresponding data but for each endpoint score are displayed in (d) (HDRS-17-sum), (e) (HDRS-6) and (f) depressed mood (f). Patients deteriorating during treatment were coded as having an endpoint fraction of 100% (a-b) or a change score of zero (c). The share of patients deteriorating was (a) duloxetine 9.1%; placebo 14.3%, (b) duloxetine 7.5%; placebo 13.7%, (c) duloxetine 3.1%; placebo 7.2%. Endpoint scores were capped at 40 points (d) and 20 points (e), respectively. The share of patients scoring above these thresholds was (d) SSRIs 0.00%, placebo 0.00%, (e) SSRIs 0.00%, placebo 0.07%. Shown is the ITT-LOCF population; n=2 168 (duloxetine) and n=1 310 (placebo).

**Supplementary figure 3** Cumulative proportions of duloxetine- and placebo-treated patients scoring on or below each 5% fraction of baseline scores at endpoint are shown for HDRS-17-sum in (a) and for HDRS-6-sum in (b). Cumulative proportions of duloxetine- and placebo-treated patients reporting different score reductions with respect to depressed mood are shown in (c). Corresponding data but for each endpoint score are displayed in (d) (HDRS-17-sum), (e) (HDRS-6) and (f) depressed mood (f). Patients deteriorating during treatment were coded as having an endpoint fraction of 100% (a-b) or a change score of zero (c). The share of patients deteriorating was (a) duloxetine 5.0%; placebo 9.6%, (b) duloxetine 4.1%; placebo 9.5%, (c) duloxetine 1.5%; placebo 5.5%. Endpoint scores were capped at 40 points (d) and 20 points (e), respectively, with no duloxetine- or placebo-treated patients scoring above these thresholds. Shown is the observed cases population; n=1 718 (SSRI) and n=989 (placebo).

.
